# Potential Cisatracurium-Induced Malignant Hyperthermia: A Case Report

**DOI:** 10.7759/cureus.76686

**Published:** 2024-12-31

**Authors:** Sinen Zeleke, Victoria L Watson, Catherine Adams, Mohammed Al-Ourani

**Affiliations:** 1 Internal Medicine, Marshall University Joan C. Edwards School of Medicine, Huntington, USA; 2 Pulmonary and Critical Care Medicine, Marshall University Joan C. Edwards School of Medicine, Huntington, USA; 3 Pulmonology and Critical Care, Marshall University Joan C. Edwards School of Medicine, Huntington, USA

**Keywords:** case report, dantrolene, malignant hyperthermia, nondepolarizing neuromuscular anesthetics, volatile anesthetics

## Abstract

Malignant hyperthermia is a pharmacogenetic disorder that manifests clinically as a hypermetabolic crisis when a patient with a mutation in the ryanodine or dihydropyridine receptor genes is exposed to neuromuscular blocking agents. Depolarizing neuromuscular agents are known to cause malignant hyperthermia, but cases caused by nondepolarizing agents are rarely reported. We present a case consistent with malignant hyperthermia after receipt of cisatracurium, a nondepolarizing anesthetic agent. A 57-year-old male patient presented with shortness of breath and increased edema of the lower extremities, found to be clinically volume overloaded. Respiratory status did not improve with diuresis and non-invasive ventilation. He developed severe acute respiratory distress syndrome necessitating endotracheal intubation and ventilation. The patient was beginning to clinically recover when he went into several instances of cardiopulmonary arrest with the eventual return of circulation. An intravenous infusion of cisatracurium was initiated to improve ventilation, and the patient’s core temperature rose to 109 degrees F shortly thereafter. Dantrolene was given with an improvement in temperature just before the patient’s family opted for comfort measures. Based on our analysis using the Naranjo score for adverse drug reactions, cisatracurium was the probable culprit for the development of malignant hyperthermia. The development of malignant hyperthermia in response to non-depolarizing neuromuscular blockers is a very rare phenomenon but should remain on the list of differential diagnoses in the setting of rapid rise in temperature so that dantrolene may be administered as quickly as possible.

## Introduction

Individuals who suffer from malignant hyperthermia generally have mutations in their ryanodine receptor subtype 1 (RYR1) or dihydropyridine (DHP) receptor genes for skeletal muscles. Contraction of the muscles without this genetic mutation is facilitated by the release of calcium from the sarcoplasmic reticulum via the RYR1 into the intracellular space, and subsequent reuptake of calcium by the sarcoplasmic reticulum leads to muscle relaxation. In the setting of genetic mutations for malignant hyperthermia and a triggering agent, calcium release is unregulated and leads to accumulation, causing prolonged muscular contraction. This process results in increased metabolic rates, leading to tachycardia and hypercarbia, which are the earliest clinical signs of malignant hyperthermia. The rigidity of muscles can be seen early in the clinical course at times, with hyperkalemia and myoglobinuria as a result of rhabdomyolysis occurring later in the disease course. Later in the course of the condition, patients rapidly develop severe hyperthermia leading to widespread organ dysfunction [1].

Triggers for the development of malignant hyperthermia are volatile inhaled anesthetics and depolarizing neuromuscular blocking agents such as succinylcholine. It is generally accepted that non-depolarizing neuromuscular blocking agents do not cause the development of malignant hyperthermia, as they are thought to be protective against malignant hyperthermia due to the blockage of excitation-coupled calcium entry [2]. Dantrolene is the only known reversal agent for malignant hyperthermia and exerts its effect by binding to the RYR1 in order to stop unregulated release of calcium [1]. Treatment with dantrolene must begin emergently to improve rates of morbidity and mortality [3].

In this case report, we will discuss the rare occurrence of malignant hyperthermia with the use of cisatracurium, a non-depolarizing agent.

## Case presentation

**Figure 1:**
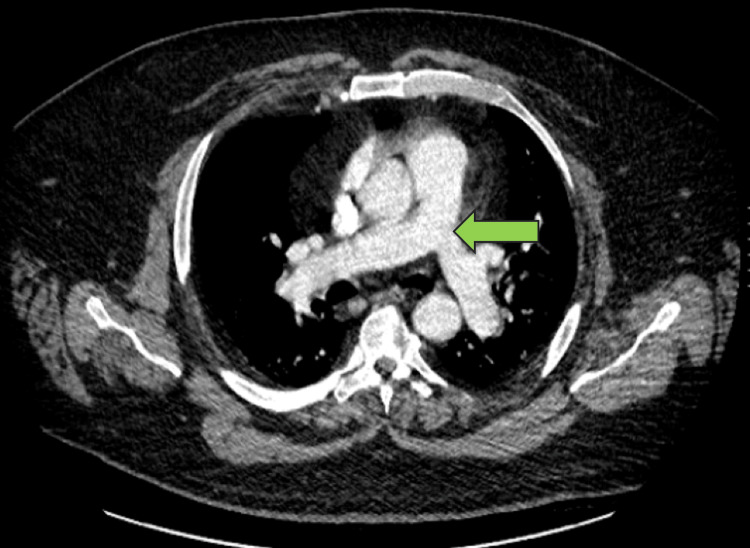
CT chest pulmonary embolism protocol on the day of admission, demonstrating patent pulmonary arteries as shown by the green arrow.

A 57-year-old male patient presented to the emergency department via ambulance with shortness of breath and worsening lower extremity edema, and was found to have an oxygen saturation of 80% on room air via pulse oximeter. His past medical history was significant for hypertension and morbid obesity with a body mass index of 52.32 kg/m^2^, without a prior history of lung disease. He also had no history of prior intubation or general anesthesia events. He was afebrile at the time of admission. Arterial blood gas showed pH 7.29/partial pressure of carbon dioxide (PaCO2) 85/partial pressure of oxygen (PaO2) 36/bicarbonate (HCO3) 32. All infectious workups were negative, including SARS-CoV-2 (COVID-19). Troponins were within normal range. Chest radiography demonstrated pulmonary congestion and cardiomegaly. Chest CT with pulmonary embolism (PE) protocol was negative for pulmonary embolus, infiltrates, congestion, and effusions (Figure 1).

The patient was admitted to the intensive care unit (ICU), diuresed using intravenous (IV) furosemide, started on empiric antibiotics for possible pneumonia, started on IV glucocorticoids, and placed on non-invasive positive pressure ventilation. His clinical picture was consistent with severe acute respiratory distress syndrome (ARDS) and required constant non-invasive ventilation for about 24 hours without significant improvement in clinical status or arterial blood gas despite concurrent diuresis. Thus, the decision was made to intubate and mechanically ventilate using rocuronium once as a part of rapid sequence intubation. The patient started to improve on mechanical ventilation after a week of care in the ICU, was following commands, and tolerating spontaneous breathing trials.

After several days of vital signs being within normal range, on the morning of ICU day 8, the patient suddenly developed hypoxia and bradycardia leading to loss of pulse while being repositioned in bed by nursing staff during routine care. Advanced Cardiovascular Life Support (ACLS) protocol was initiated, and return of spontaneous circulation (ROSC) was obtained after epinephrine and defibrillation. Chest radiography and lab work obtained during this arrest episode were similar to the previous one, with the exception of worsening lactic acidosis. Hypoxia did not improve with bag-mask ventilation via endotracheal tube, and the patient lost pulse again. ACLS was again initiated, and ROSC was again obtained. Rocuronium was also given as a single dose due to high positive end-expiratory pressure (PEEP) requirements and persistently low oxygen saturations, with subsequent improvement in oxygenation status.

The patient’s condition stabilized for about 14 hours until he developed worsening hypoxia later in the day. Since a one-time dose of rocuronium improved hypoxia in the previous code event, the decision was made to start a cisatracurium bolus followed by continuous infusion. Approximately two hours after the bolus and beginning of the infusion, the patient's esophageal temperature probe measured 108.6 degrees F, whereas it had previously been around 102 degrees F. Examination was significant for muscle rigidity, and labwork demonstrated increased PaCO2 and lactic acid. Creatine kinase (CK) was elevated to 350 U/L, and potassium was elevated to 5.2 mEq/L. Due to the rapid rise and degree of temperature elevation and lab findings, malignant hyperthermia was suspected and the cisatracurium was stopped immediately. The Naranjo score for adverse drug events totaled 5 (Table 1). A loading dose of 372.5 mg of dantrolene was administered, with a minor improvement in temperature of about 1.4 degrees F. A repeat dose of dantrolene was also given; however, the patient subsequently arrested again and the family opted to pursue comfort care.

**Table 1:** Naranjo score for adverse drug events calculated with total score of 5.

Question	Yes	No	I don’t know	Our score
Are there any previous conclusive case reports on this reaction?	+1	0	0	0
DId the adverse event appear after the suspected drug was administered?	+2	-1	0	+2
Did the adverse event improve when the drug was discontinued or a specific antagonist was administered?	+1	0	0	+1
Did the adverse event reappear when the drug was readministered?	+2	-1	0	0
Are there alternative causes that could on their own have caused the reaction?	-1	+2	0	+2
Did the reaction reappear when a placebo was given?	-1	+1	0	0
Was the drug detected in blood or other fluids in concentrations known to be toxic?	+1	0	0	0
Was the reaction more severe when the dose was increased or less severe when the dose was decreased?	+1	0	0	0
Did the patient have a similar reaction to the same or similar drugs in any previous exposure?	+1	0	0	0
Was the adverse event confirmed by any objective evidence?	+1	0	0	0
Total				5

On admission, a chest CT scan with PE protocol was negative for PE. However, following the code event, the patient remained too unstable to undergo repeat chest imaging for further evaluation of PE. Additionally, the onset of acute kidney injury contraindicated the use of contrast dye required for another chest CT scan. Given these limitations and the clinical suspicion for PE, empiric heparin infusion was initiated during the code events.

## Discussion

Malignant hyperthermia is defined as a hypermetabolic response of skeletal muscle, typically to volatile inhaled anesthetics such as halothane, isoflurane, desflurane, and sevoflurane, or depolarizing neuromuscular agents such as succinylcholine [1,2]. A mechanism for the development of malignant hyperthermia with non-depolarizing neuromuscular agents is not known.

In this case, the patient did not receive succinylcholine or other depolarizing neuromuscular agents at any point during his admission. However, non-depolarizing agents were used. Rocuronium was administered to this patient on two occasions: once for endotracheal intubation, seven days prior to the arrest events and development of malignant hyperthermia, and once while intubated, about 14 hours prior to the arrest events and development of malignant hyperthermia. Between the administration of rocuronium and the cardiac arrests, the temperature remained stable at approximately 100 degrees F. A study by Beggs et al. describes two patients who developed malignant hyperthermia with rocuronium; however, they noted that this developed only with continuous infusion of the drug, which was not the case for our patient [4].

There are two reported cases of cisatracurium-induced malignant hyperthermia available in the existing literature. In the first, a 60-year-old male patient who tested positive for SARS-CoV2 developed worsening respiratory status requiring intubation and paralysis for ventilator compliance [5]. Sixteen hours after cisatracurium infusion was initiated, the patient’s core temperature rose to 106.6 degrees F. A one-time dose of dantrolene 250 mg was administered, and decreased the patient’s temperature to 101.5 degrees F over a four-hour period. Another case report, which was presented at the 2019 Chest conference, also involved a 57-year-old male patient with respiratory distress; in this case, after a motor vehicle accident [6]. His respiratory status ultimately worsened, necessitating intubation. Cisatracurium was used for paralysis and was continued as an infusion during mechanical ventilation. The patient’s core temperature climbed to 106 degrees F and recovered rapidly with the administration of one dose of dantrolene. As pointed out by Sathyanarayanan et al. [5], it is curious that two reports of cisatracurium-induced malignant hyperthermia occurred in the setting of ARDS [5,6]. This is also true for our current case, further suggesting a possible link between ARDS and the development of malignant hyperthermia upon exposure to cisatracurium.

Masseter muscle rigidity has been noted as a risk factor for the development of malignant hyperthermia. Vecuronium leading to masseter muscle rigidity has also been reported, as have other non-depolarizing neuromuscular agents [7,8]. Studies performed on pigs susceptible to malignant hyperthermia revealed that vecuronium, doxacurium, and mivacurium were not triggers for malignant hyperthermia [9,10]. Another study, also performed in susceptible pigs, suggested that pancuronium delayed the onset of malignant hyperthermia in combination with inhaled halothane anesthetics [11].

In the present case, it was only after cisatracurium had been infused for at least one hour that the temperature began to rise rapidly and to levels rarely seen with infection alone, making cisatracurium the more likely culprit for the development of malignant hyperthermia. When the Naranjo scale was used to determine the likelihood that this adverse drug reaction was attributable to the drug rather than other factors, we found a score of 5, which suggests that cisatracurium is the probable culprit.

The current case study is limited by the lack of a definitive diagnosis in a severely ill patient. Due to the acute onset of the condition, the severity of the patient’s illness, and the family’s choice to pursue comfort measures, the confirmatory tests for malignant hyperthermia such as the Caffeine Halothane Contracture Test (CHCT) via muscle biopsy, and genetic testing, were not obtained. The CHCT has a reported low false negative rate of 97%, thus effectively ruling out malignant hyperthermia. However, this test has about a 20% rate of false positives, requires surgical muscle biopsy, as well as a several-month waiting period after any event concerning for potential malignant hyperthermia [12]. Genetic testing via blood sample for the most common RYR1 mutations on chromosome 19 is much more convenient for patients. This method also has false negatives, as not all mutations causing malignant hyperthermia have yet been identified [13].

Other lab studies such as elevated potassium and CK levels can suggest the potential development of malignant hyperthermia. The presented patient did have low potassium levels requiring repletion in the days prior to the code events and development of malignant hyperthermia, with potassium increase to 5.2 mEq/L surrounding the code events/development of malignant hyperthermia. It is also possible that another concurrent diagnosis such as pneumonia, translocation of gut bacteria from the code event, or even hypothalamic damage could account for high fevers, and the myoclonic activity due to cerebral hypoxia from the code events could have led to increased rigidity. However, while the possibility of propofol-related infusion syndrome could not be entirely excluded, the rapid development of cisatracurium-induced malignant hyperthermia is a more likely diagnosis in this clinical context, though the evidence available in our case does not make a definitive diagnosis.

## Conclusions

The development of malignant hyperthermia in response to non-depolarizing neuromuscular agents is a very rare phenomenon but should remain on the list of differential diagnoses in the setting of a rapid rise in temperature. Timely diagnosis and treatment are essential to avoid mortality. In the case presented in this report, the patient's family opted to pursue comfort care given a complicated hospital course, and the patient ultimately expired.
